# The Evolution of Patient-Centered Nursing Care: From Passive Recipients to Active Partners in the Era of Generative AI

**DOI:** 10.1097/jnr.0000000000000765

**Published:** 2026-08-13

**Authors:** Fei-Hsiu Hisao

**Affiliations:** School of Nursing, College of Medicine, National Taiwan University; and Department of Nursing, National Taiwan University Hospital, Taipei, Taiwan, ROC

Nurses have traditionally played an active role in providing patients with knowledge about health, disease, and self-care based on the assumption that patients lack sufficient knowledge and are unable to acquire it independently. Today, the widespread accessibility of artificial intelligence (AI) technologies has made health-related information readily available to the public, enhancing the autonomy and learning capabilities of patients and facilitating the transformation of the nursing care paradigm. As patients become more informed and engaged, their roles are shifting from passive recipients of care to active partners in collaboration with healthcare professionals.

Mesko et al. (2025) reviewed the 25-year evolution of patient empowerment and examined its impact on patient–physician relationships, highlighting a movement toward greater partnership and improved health outcomes. Advances in digital technologies have empowered patients by enabling them to access the information they need independently. Digital health tools such as wearable sensors support the ability of patients to monitor their own health and proactively engage in early disease management. Furthermore, generative AI applications help patients enhance their health knowledge, better understand their medical conditions, and make informed decisions regarding treatment and care. This review also demonstrated that empowered patients who actively participate in shared decision-making processes experience improved health outcomes and greater satisfaction with care.

A concept analysis of AI-assisted nursing care by Nematollahi et al. (2025) highlights the growing role of AI-assisted nursing care within the rapidly evolving healthcare environment. The integration of AI into personalized and precision nursing care includes AI-driven systems that analyze patient data, detect health problems in a timely manner, and provide nurses with evidence-based recommendations. The reported benefits of AI-assisted nursing care include improved patient outcomes, increased efficiency in nursing practice, enhanced nurse satisfaction, and reduced healthcare costs.

This issue of *The Journal of Nursing Research* highlights several important topics, including enhancing intrinsic motivation among patients with schizophrenia to improve medication adherence and health outcomes; addressing health literacy among patient–family dyads; strengthening nurses’ participation in patient decision-making; and increasing nurses’ awareness of attitudes toward lesbian, gay, bisexual, transgender, and queer (LGBTQ) individuals. In this new era of patient-centered nursing care, nurses play a critical role in empowering patients and families to engage in autonomous learning using generative AI technologies. Nurses can further support the delivery of personalized and precision nursing care by considering the unique learning experiences, varying levels of health literacy, and diverse gender-related needs of their care recipients.
